# Editorial: The interplay between oxidative stress, immune cells and inflammation in cardiovascular diseases

**DOI:** 10.3389/fcvm.2024.1385809

**Published:** 2024-02-27

**Authors:** R. Nosalski, M. Siedlinski, K. B. Neves, C. Monaco

**Affiliations:** ^1^ Centre for Cardiovascular Sciences, University of Edinburgh, Edinburgh, United Kingdom; ^2^ Department of Internal Medicine, Faculty of Medicine, Jagiellonian University Medical College, Krakow, Poland; ^3^ Omicron Medical Genomics Laboratory, Jagiellonian University, Collegium Medicum, Krakow, Poland; ^4^ Strathclyde Institute of Pharmacy and Biomedical Sciences, University of Strathclyde Glasgow, Glasgow, United Kingdom; ^5^ Kennedy Institute of Rheumatology, NDORMS, University of Oxford, Oxford, United Kingdom

**Editorial on the Research Topic** The interplay between oxidative stress, immune cells and inflammation in cardiovascular diseases

Cardiovascular diseases (CVDs) constitute the leading causes of death and reduced quality of life worldwide. Reactive oxygen species (ROS), various immune cells and cytokines participate in crucial signalling pathways implicated in the homeostasis of the cardiovascular system. Dysregulated ROS production and immune system activation have been widely demonstrated to lead to the development of CVDs, hypertension, target organ damage and cardiovascular complications (1, 2). Hypertension is a major risk factor leading to the development of cardiovascular diseases and target organ damage, including hypertensive heart disease. This pathology leads to abnormalities of the heart, involving changes in the structure and function of the left ventricle, the left atrium and coronary arteries. Masenga and Kirabo nicely summarised the current knowledge about hypertensive heart disease, highlighting this cardiac pathology's mechanisms, complications, and implications. In their comprehensive review, published in the current issue, they focused specifically on left ventricular hypertrophy, atrial fibrillation, heart failure, and coronary heart disease, and current and future therapies targeting these complications.

Target organ damage might be accelerated by excess salt intake, which activates NADPH oxidases and the formation of toxic lipid peroxidation products, isolevuglandins, which possess direct biological activity through modification of proteins' biological properties. Ferroptosis is a form of regulated cell death that is characterised by the iron-dependent accumulation of lipid peroxides. It is distinct from other forms of cell death, such as apoptosis, necrosis, and autophagy, and we know less about it. Ferroptotic cells and isolevuglandin protein adducts can be presented by dendritic cells or macrophages to T cells, which, upon activation, produce various pro-inflammatory cytokines (e.g., TNF-α, IL-6 and IL-1β) that contribute to the development and propagation of CVDs (3). In the current research topic, Wang and Wu reviewed the recent findings, which may provide a better understanding of the contribution of ferroptosis in the pathogenesis of CVDs. Additionally, the review provides valuable insights into the potential therapeutic strategy utilising ferroptosis inhibitors or modulation of ferroptosis-related non-coding RNAs for targeting CVDs.

In immune cells, high uric acid is shown to promote ferroptosis of immune cells. Uric acid crystals can accumulate in target organs, leading to the development of systematic inflammation, activation of immune cells and induce oxidative stress (4, 5). Burnier discussed in the current research topic the role of gout and hyperuricaemia and their epidemiological and genetic associations with CVDs. Both clinical conditions may promote cardiovascular complications. And this is especially important because uric acid has been suggested as an independent risk factor for cardiovascular events such as hypertension (6). However, the treatment for asymptomatic hyperuricaemia isn't standard these days, unless uric acid level is significantly elevated. The author encouraged lowering uric acid thresholds to prevent the development of cardiovascular complications using currently available therapies such as xanthine oxidase inhibitors or novel therapeutic approaches targeting SGLT2. However, this should be further investigated in randomised clinical trials and prospective studies.

Various risk scores that include haematological parameters and inflammatory biomarkers are calculated based on neutrophil, lymphocyte, monocyte, and platelets counts. Numerous studies have shown the prognostic value of neutrophil-to-lymphocyte ratio (NLR), monocyte-to-lymphocyte ratio (MLR), platelet-to-lymphocyte ratio (PLR), and systemic immune-inflammation index (SII) in various CVDs (7–9). In the current issue, Wu et al. conducted a retrospective analysis using publicly available data from the NHANES (1999–2018) to investigate the relationship between the pan-immune inflammation value (PIV), which incorporates the counts of neutrophils, platelets, monocytes, and lymphocytes with long-term mortality in hypertensive patients. In their study, all-cause mortality and cardiovascular mortality were significantly elevated among participants with the highest PIV levels. Furthermore, Cox regression analysis showed that a high PIV level was an independent risk factor for long-term mortality in hypertensive patients. Interestingly, the evaluation of the immune and inflammatory status using PIV has been shown to be a better predictor of cardiovascular outcomes than other inflammatory markers (7, 9).

The current issue closes with an original work provided by Sulicka-Grodzicka et al. and their investigation of whether levels of circulating TNF-α, IL-6 and IL-1β reflect their local expression in the blood vessels of patients with advanced coronary disease. The authors used coronary arteries, an essential site of atherosclerosis, and internal mammary arteries, which are resistant to the development of this pathology. However, both develop endothelial dysfunction, which is a hallmark of CVDs. Although TNF-α, IL-6 and IL-1β have already been widely linked to vascular diseases, such as atherosclerosis and hypertension (10), the authors did not find any correlations between the systemic level and vascular mRNA expression of these pro-inflammatory cytokines. Additionally, the expression of TNF-α, IL-6 and IL-1β was not associated with enhanced superoxide production observed in the internal mammary arteries collected from patients undergoing coronary artery bypass graft (CABG) surgery. Despite various limitations of the study, the work highlights a mechanistic disconnect between systemic and local inflammation, the former linked to systemic stimuli linked to cross-organ regulation and the latter regulated by local factors. Further studies are needed to identify the role of local inflammation and activation of NADPH oxidase within the perivascular niche.

Overall, this research topic emphasised the role of oxidative stress and inflammation in a broad range of CVDs and new risk factors emerging in the field. It provides insights into the role of ferroptosis, gout and hyperuricaemia in the pathogenesis of CVDs and target organ damage. Finally, this collection highlights the importance of searching for novel biomarkers reflecting local inflammation and the need for large-scale retrospective studies to develop novel risk scores in order to improve the prevention of CVDs.
